# Association between provegetarian food patterns and micronutrient adequacy in preschoolers: the SENDO project

**DOI:** 10.1007/s00431-024-05808-9

**Published:** 2024-10-07

**Authors:** Elise Fabios, Itziar Zazpe, Miguel Ángel Martínez-González, Mónica Arias, Esther Ruiz-Chércoles, Nerea Martín-Calvo

**Affiliations:** 1 Department of Preventive Medicine and Public Health, School of Medicine, University of Navarra, 31008 Pamplona, Spain; 2 IdiSNA, Instituto de Investigación Sanitaria de Navarra, 31008 Pamplona, Spain; 3 Biomedical Research Centre Network On Obesity and Nutrition (CIBERobn), Physiopathology of Obesity and Nutrition, Institute of Health Carlos III, 28029 Madrid, Spain; 4 Department of Nutrition, Food Science and Physiology, School of Pharmacy, University of Navarra, 31008 Pamplona, Spain; 5 School of Nursing, University of Navarra, Pamplona, Spain; 6 Health Care Centre María Jesús Hereza, Jesus Miguel Haddad Blanco 2, 28911 Leganés, Madrid Spain

**Keywords:** Flexitarian diet, Healthful provegetarian diet index, Micronutrient adequacy, Provegetarian food patterns score, SENDO cohort

## Abstract

This study aims to examine the association between different versions of a provegetarian food pattern and micronutrient adequacy in a population of children from the SENDO Project. Children aged 4–5 years old were recruited and baseline information on diet, lifestyle and socio-demographic characteristics was collected through self-administered online questionnaires completed by their parents. Scores were calculated for overall, healthful, and unhealthful provegetarian food patterns (FP) using an a priori approach. Participants were categorized into tertiles according to their scores. Micronutrient adequacy was assessed using the Estimated Average Requirement (EAR) cut-off point. Multivariate analyses were performed to evaluate the relationship between tertiles of each provegetarian FP and the risk of inadequate micronutrient intake (failing to meet ≥ 3 requirements). Despite lower intakes of certain micronutrients, children with higher scores in the healthful provegetarian FP did not exhibit a higher prevalence of inadequacy. Children in the highest tertile of this index had 0.47-fold lower odds (95%CI 0.23–0.95) of having ≥ 3 inadequate micronutrient intakes than their peers in the lowest tertile, after adjusting for potential confounders. In contrast, children in the highest tertile of the unhealthful provegetarian FP had 20.06-fold higher odds (95%CI 9.19–43.79) of having ≥ 3 inadequate micronutrient intakes compared to children in the lowest tertile.

*Conclusions*: Adherence to a healthful provegetarian food pattern is associated with improved nutritional adequacy in preschoolers, while following an unhealthful pattern is detrimental to micronutrient adequacy. These results suggest that moderate reductions in animal food consumption within a healthy diet may not compromise micronutrient adequacy in young children. Further research is needed to explore the impact of diets reduced in animal food intake on the health of children.

**Table:** 

**What is Known:** • *There's a notable trend towards plant-based diets due to health and sustainability concerns.* • *Research links plant-based diets in adults with lower risks of obesity, cardiovascular disease, and cancer.*
**What is New:** • *A new study examines how different versions of a provegetarian food pattern affect micronutrient adequacy in children, using a moderate and stepwise approach.* • *Following a healthful provegetarian food pattern enhances nutritional adequacy in preschoolers, whereas an unhealthful pattern negatively impacts micronutrient adequacy.*

**Supplementary Information:**

The online version contains supplementary material available at 10.1007/s00431-024-05808-9.

## Introduction

In recent years, increasing sectors of the population are shifting to dietary patterns that restrict animal products, motivated by health considerations and other concerns related to the sustainability of the environment, animal welfare or religious reasons. In adults, plant-based diets such as vegetarian or vegan diets have been associated with a lower risk of obesity, cardiovascular disease, and cancer, and from an environmental perspective with a smaller ecological impact [1–4].

There is some concern as to whether diets restricted in animal-derived foods can fulfill nutritional requirements in childhood and adolescence, due to the increased needs related to rapid growth and development. However, evidence is still scarce, and debate is ongoing regarding the nutritional adequacy of dietary patterns with high or total restriction of animal-derived foods during childhood [5–9].

Moreover, not all plant-based diets fall under the categories of vegetarian or vegan [10, 11]. In general, plant-based diets emphasize plant products, such as wholegrains, fruits, vegetables, nuts, legumes and seeds, and limit or avoid animal products. But the amount and type of animal food restriction can vary substantially among different plant-based diets. Some authors have referred to those more flexible patterns as flexitarian, semi-vegetarian or provegetarian, but the wide spectrum of plant-based dietary patterns, with varying degrees of animal restriction, is difficult to encompass with simple definitions [10].

Thus, Martínez-González et al. proposed a provegetarian food pattern (FP) score, that weights positively plant-based foods and negatively animal-derived foods [12]. Satija et al. later developed two additional scores, a healthful and an unhealthful plant-based diet index, to account for differences in dietary choices, as not all “provegetarian” FP are necessarily healthy [13]. Recent evidence has shown that the overall provegetarian FP score and its healthful version are associated with favorable health outcomes in adults, such as lower risk of nutritional inadequacy [14], overweight and obesity [15], cardiometabolic disease [13, 16, 17], diabetes [4], specific cancers [18], and all-cause mortality [12, 16, 19].

To date, there is no study on provegetarian FPs and children’s health. Since the question of nutrient adequacy in children consuming restrictive diets is a longstanding controversy, it would be important to analyze the question under the lens of a moderate and stepwise approach, as provided by the provegetarian FP score conceptual framework [12].

Therefore, this study aims to investigate the association between different versions of a provegetarian FP (overall, healthful, and unhealthful) and micronutrient adequacy, in a population of children from the SENDO Project *(Seguimiento del Niño para un Desarrollo Óptimo).*

## Material and methods

### Study population

The SENDO Project is a dynamic, prospective cohort of Spanish children that began recruiting participants in 2015 (https://www.proyectosendo.es/). Its primary aim is to assess the effect of lifestyle and diet on the health of children and adolescents. Participants are invited to join the cohort by their pediatrician at their health care center or by the SENDO team of researchers through schools. Inclusion criteria are being a child 4 or 5 years old and residing in Spain. The sole exclusion criterion is the impossibility to access a device connected to the internet to fulfill the online questionnaires. Self-reported questionnaires are completed online by the child’s parents and collected at baseline and updated every year. The baseline questionnaire collects information related to lifestyle, diet, medical history, anthropometric and sociodemographic variables.

This cross-sectional study is based on baseline information of participants recruited between January 2015 and June 2023. A total of 1208 participants were recruited. We excluded 141 participants who reported extreme energy intake (< P1 or > P99) and 118 with extreme micronutrient intakes (≥ + 3 or ≤ -3 standard deviations (SD) from the mean). Lastly, 85 participants were additionally excluded for presenting incomplete questionnaires at baseline. Thus, the final sample included 864 participants.

### Ethical approval

The SENDO project adheres to the rules of the Declaration of Helsinki on the ethical principles for medical research in human beings. This study was approved by the Ethics Committee for Clinical Research of Navarra (P. 2016/122). An informed consent was obtained during the recruitment from all participants’ parents.

### Dietary Assessment

The usual dietary intake information was collected through a validated semi-quantitative 147-item food-frequency questionnaire (FFQ) [20] completed by the parents.

### Provegetarian FP Scores

We calculated adherence to a provegetarian FP based on three scores: an overall provegetarian FP score [12] and two additional ones, that distinguish between healthful and unhealthful plant-based foods (healthful provegetarian and unhealthful provegetarian FP scores) [13]. The scoring criteria for all three scores are shown in Table 1 and has been described in previous studies [4, 12, 13, 15].

**Table 1:** Scoring criteria for the provegetarian FPs

Overall provegetarian FP^1^ (potential range 12–60)		Healthful/unhealthful provegetarian FP^2^ (potential range of 17–85)		
Component	Criteria	Component	Criteria	
			Healthful	Unhealthful
**Plant food groups**		**Plant food groups**		
		***Healthful***		
1. Vegetables	Positive	1. Vegetables	Positive	Reverse
2. Fruits	Positive	2. Fruits	Positive	Reverse
3. Legumes	Positive	3. Legumes	Positive	Reverse
4. Cereal grains	Positive	4. Whole grains	Positive	Reverse
5. Potatoes	Positive	5. Nuts	Positive	Reverse
6. Nuts	Positive	6. Olive oil	Positive	Reverse
7. Olive oil	Positive			
		***Less healthful***		
**Animal Food Groups**		7. Fruit juices	Reverse	Positive
8. Dairy products	Reverse	8. Potatoes	Reverse	Positive
9. Eggs	Reverse	9. Refined grains	Reverse	Positive
10. Meat	Reverse	10. Sugary beverages	Reverse	Positive
11. Fish and seafood	Reverse	11. Pastries	Reverse	Positive
12. Animal fat	Reverse			
		**Animal Food Groups**		
		12. Dairy products	Reverse	Positive
		13. Eggs	Reverse	Positive
		14. Meat	Reverse	Positive
		15. Fish and seafood	Reverse	Positive
		16. Miscellaneous food^3^	Reverse	Positive
		17. Animal fat	Reverse	Positive

*FP* Food Pattern

^1^The overall provegetarian FP score was built by summing both components with a potential range of 12–60

^2^The healthful and unhealthful provegetarian FP scores were built by summing both components with a potential range of 17–85

^3^Miscellaneous food includes pizza, lasagna, instant soup, mayonnaise, croquettes, nuggets, snacks, popcorn

For the analysis of the overall provegetarian FP, we included 7 different plant food groups (fruits, vegetables, potatoes, nuts, legumes, grains, and olive oil) and 5 animal food groups (dairy, eggs, meat, fish and seafood, and animal fat), as originally proposed [13]. Food consumption (g/d) was adjusted for total energy intake by using the residual method. The energy-adjusted estimates were ranked into quintiles (for plant-based food groups) and reverse quintiles (for animal food groups). For plant-based food groups, a value of 1 was assigned to the first quintile, 2 to the second quintile, and so on, until 5 was assigned to the fifth quintile. For animal products quintiles were reversed (assigning a value of 5 to the first quintile, 4 to the second quintile, and so on, until a value of 1 was assigned to the fifth quintile). Final score ranged from 12 (lowest adherence) to 60 (highest adherence).

The healthful and unhealthful scores were constructed according to the criteria exposed by Satija et al. [4], by dividing plant-food groups according to their impact on health. The analysis included 17 items for both scores. Unlike the original index designed for adults, the item 'tea/coffee’ was not included in the score since our study focused on children. Food consumption was also adjusted for total energy intake (residual method) [21]. For the healthful provegetarian FP, we assigned quintile values for healthful plant food consumption and reverse quintiles values for animal food consumption and unhealthful plant food consumption. For the unhealthful provegetarian FP, we assigned quintile values for unhealthful plant food consumption and reverse quintiles values for animal food consumption and healthful plant food consumption. Final scores for both indexes ranged from 17 (lowest adherence) to 85 (highest adherence).

We also analyzed dietary intake through 16 different food groups: vegetables, fruits, legumes, grains, nuts, olive oil, potatoes, fruit juices, dairy, eggs, fish and seafood, meat, animal fat, sugar-sweetened beverages, pastries and sweets, beverages, and miscellaneous. Lastly, we separately assessed adherence to the Mediterranean diet by using the KIDMED score, which has been described previously [22].

### Covariates

The participants’ sociodemographic information was reported by their parents. The questionnaire on physical activity collected information on 14 types of activities, with 10 different possible answers, ranging from never to ≥ 11 h per week. Participants indicated the average time dedicated to each activity in the previous year. Screen time was assessed by averaging the daily hours spent using screens (TV, computer, or video games). Time spent on weekdays and weekends was assessed separately.

Parental attitudes towards their child’s dietary habits were assessed with 8 yes/no questions, scored positively for healthy attitudes and given no point for unhealthy ones. Parental knowledge on dietary recommendations for children were evaluated with questions on the recommended intake frequency of 18 food groups. Both scores have been described in previous articles [23, 24]

### Outcome assessment

We assessed the intake of 20 micronutrients, including vitamins A, C, D, E, B1, B2, B3, B6, B12, folic acid, Calcium (Ca), Iodine (I), Iron (Fe), Phosphorous (P), Magnesium (Mg), Selenium (Se), Zinc (Zn), Chromium (Cr), Potassium (K) and Sodium (Na). Participants were considered to have an inadequate micronutrient intake when their intake was inferior to the estimated average requirement (EAR), (or to the adequate intake (AI) if the EAR was not available), as established by the Institute of Medicine [25].

### Statistical analysis

Participants were divided into tertiles according to their provegetarian FP scores, with the highest tertile representing higher adherence and the lowest tertile representing lower adherence. Participants’ sociodemographic characteristics were presented according to their adherence to the overall provegetarian FP and information on nutrient and food group intake was presented according to adherence to all three provegetarian FPs. Numbers and percentages were used for categorical variables and means and SD were used for continuous variables. Linear trend tests across tertiles of each score were calculated by assigning the median of each tertile and treating this variable as continuous in regression models. We also assessed the prevalence of inadequate intake of each micronutrient in each tertile of the three patterns.

Multivariate analyses were used to assess the relationship between each provegetarian FPs and the risk of having ≥ 3 inadequate intakes, by using the EAR cut-point method. We fitted generalized estimating equations to account for intra-cluster correlation between siblings.

Analyses were progressively adjusted, in different models, for the following potential confounding factors: 1) age (continuous), sex, and total energy intake (continuous); 2) breastfeeding duration (none, < 6 months, 6–12 months, and > 12 months), number of siblings (1, 2, 3–4, 5 or more), parental knowledge about nutritional recommendations for children (low, medium or high) and parental attitudes towards child’s dietary habits (unhealthy, average or healthy); 3) physical activity (continuous) and screen time (continuous). The first tertile was used as category of reference.

For our analysis, we calculated 1) the number of micronutrients with inadequate intake (and 95% Confidence Interval (CI)) by tertiles of provegetarian scores and 2) the Odds Ratio (OR) and 95% CI for the inadequate intake of ≥ 3 micronutrients across tertiles of the three provegetarian FPs. We also estimated the adjusted proportions of children with inadequate intake of ≥ 3 micronutrients in each tertile of the three patterns.

Lastly, different sensitivity analysis were performed to assess the robustness of our findings: 1) we included the intake of supplements to the total intake of micronutrients calculated for each participant; and 2) we changed the outcome to an inadequate intake of ≥ 4 micronutrients.

Statistical analyses were carried out using Stata version 15.0 (Stata Corp., College Station, TX, USA). All p values are two-tailed and statistical significance was settled at the conventional cut-off point of *p* < *0.05.*

## Results

This cross-sectional study included 864 participants (50.5% girls) with an average age of 5.0 years (SD: 0.8). The participants’ main characteristics according to tertiles of the overall provegetarian FP index are presented in Table 2.

**Table 2:** Characteristics of participants and their families in the SENDO project by tertiles of the overall provegetarian score. Numbers are mean (SD) or *N* (%)

	T1	T2	T3	*p* for trend
***n***	354	228	282	
**Range of Provegetarian Score**	19–33	34–38	39–53	
**Age (years)**	5.0 (0.9)	5.0 (0.8)	4.9 (0.8)	0.198
**Birthweight (g)**	3252 (567.0)	3177 (574.5)	3269 (483.8)	0.786
**Z-score of the BMI**	0.11 (1.13)	-0.03 (1.18)	0.05 (1.14)	0.454
**Moderate-vigorous physical activity (h/day)**	1.03 (0.75)	1.20 (0.83)	1.17 (0.75)	0.023
**Screen time (hours/day)**	1.15 (0.85)	1.16 (0.94)	1.01 (1.06)	0.072
**Sex (female), %**	179 (50.6)	106 (46.5)	143 (50.7)	0.979
**Race (white), %**	339 (95.8)	219 (96.1)	269 (95.4)	0.831
**Gestational age (weeks), %**				0.773
< 38	52 (14.8)	33 (14.5)	32 (11.4)	
38 to 40	131 (37.3)	97 (42.5)	118 (42.1)	
> 40	168 (47.9)	98 (43.0)	130 (46.4)	
**Birthweight (g), %**				0.587
< 2500	35 (10.0)	24 (10.5)	19 (6.8)	
2500–3000	62 (17.7)	56 (24.6)	63 (22.5)	
3000–3500	140 (39.9)	88 (38.6)	116 (41.4)	
3500–4000	89 (25.4)	53 (23.2)	67 (23.9)	
> 4000	25 (7.1)	7 (3.1)	15 (5.4)	
**Breastfeeding duration (months), %**				< 0.001
No breastfeeding	67 (18.9)	43 (18.9)	33 (11.7)	
< 6	114 (32.2)	60 (26.3)	66 (23.4)	
6 to 12	95 (26.8)	66 (28.9)	62 (22.0)	
> 12	78 (22.0)	59 (25.9)	121 (42.9)	
**Nutritional Status (g), %**				0.254
Low weight	47 (13.3)	39 (17.1)	45 (16.0)	
Normal weight	260 (73.4)	161 (70.6)	205 (72.7)	
Overweight/obesity	47 (13.3)	28 (12.3)	32 (11.3)	
**Suplement use (%)**	16 (4.6)	7 (3.1)	10 (3.6)	0.486
**Maternal age (years)**	39.9 (3.8)	40.1 (5.1)	39.8 (4.3)	0.756
**Maternal age (years), %**				0.797
< 35	33 (9.3)	30 (13.2)	37 (13.1)	
35–40	150 (42.4)	83 (36.4)	111 (39.4)	
40–45	140 (39.5)	83 (36.4)	102 (36.2)	
> 45	31 (8.8)	32 (14.0)	32 (11.3)	
**Family of history of obesity, %**	67 (19.2)	41 (18.1)	56 (20.0)	0.821
**Maternal high education%**	291 (82.2)	183 (80.3)	229 (81.2)	0.727
**Parental attitudes towards child’s dietary habits, %**				< 0.001
Low (< 40%)	26 (7.3)	10 (4.4)	8 (2.8)	
Moderate (40–70%)	137 (38.7)	70 (30.7)	71 (25.2)	
High (> 70%)	191 (54.0)	148 (64.9)	203 (72.0)	
**Parental knowledge about the child’s nutritional recommendations, %**				0.011
Low (< 40%)	82 (23.2)	56 (24.6)	53 (18.8)	
Moderate (40–70%)	233 (65.8)	144 (63.2)	175 (62.1)	
High (> 70%)	39 (11.0)	28 (12.3)	54 (19.1)	
**Number of children, %**				< 0.01
1	43 (12.1)	37 (16.2)	39 (13.8)	
2	177 (50.0)	111 (48.7)	173 (61.3)	
3–4	111 (31.4)	70 (30.7)	59 (20.9)	
5 or more	23 (6.5)	10 (4.4)	11 (3.9)	
**Child’s position among siblings, %**				0.498
The oldest/singletons	127 (35.9)	83 (36.4)	102 (36.2)	
2nd/3, 2nd or 3rd/4	59 (16.7)	35 (15.4)	31 (11.0)	
The youngest or beyond the fourth	168 (47.5)	110 (48.2)	149 (52.8)	

Children with higher scores (T3) had slightly higher energy intakes, had been breastfed for a longer period, and were more physically active. Their parents displayed healthier attitudes towards their child’s dietary habits and had greater knowledge regarding child nutrition. On the other hand, participants with lower scores (T1) came from more numerous families.

Regarding dietary composition, as displayed in Table 3, participants with higher adherence to the overall provegetarian FP (T3) consumed proportionally more carbohydrates, and less protein and fat. They also consumed slightly less saturated fat. Lastly, they consumed more fruits and vegetables, legumes, cereal grains, potatoes and nuts but less animal products than their peers in T1.

**Table 3:** Dietary composition according to tertiles of adherence to the overall, healthful and unhealthful provegetarian FPs. Mean (SD)

	Overall provegetarian FP		Healthful provegetarian FP		Unhealthful provegetarian FP	
	T1	T3	T1	T3	T1	T3
Score range	19–34	39–52	31–48	55–71	33–48	55–70
*n*	354	282	323	247	303	250
TEI (kcal)	1978 (475.5)	2088 (439.2)*	1929 (495.8)	2118 (443.2)*	1938 (397.5)	2136 (521.1)*
Carbohydrate intake (% of total energy)	41.8 (4.75)	45.3 (5.06)*	43.4 (4.90)	43.9 (5.44)	42.1 (4.98)	45.34 (5.06)*
Protein intake (% of total energy)	17.9 (1.90)	16.1 (1.97)*	17.6 (1.89)	16.3 (2.27)*	17.8 (2.07)	16.3 (2.06)*
Fat intake (% of total energy)	40.3 (4.92)	38.5 (5.30)*	38.9 (4.82)	39.7 (5.67)	40.1 (5.06)	38.8 (5.28)*
SFA intake (% of total energy)	0.12 (0.02)	0.10 (0.02)*	0.12 (0.02)	0.10 (0.02)*	0.11 (0.02)	0.11 (0.02)
MUFA intake (% of total energy)	0.15 (0.03)	0.16 (0.04)*	0.14 (0.03)	0.17 (0.04)*	0.16 (0.03)	0.14 (0.04)*
PUFA intake (% of total energy)	0.05 (0.01)	0.05 (0.01)	0.05 (0.01)	0.05 (0.01)*	0.05 (0.01)	0.05 (0.01)*
Fibre intake (g/d)	17.6 (4.91)	25.5 (6.38)*	17.3 (5.17)	26.1 (6.28)*	22.8 (6.47)	18.8 (5.91)*
KIDMED score (p50 (IQR))	6.01 (2.00)	7.88 (1.78)*	5.71 (1.96)	8.09 (1.78)*	7.70 (1.78)	5.62 (1.88)*
**Food groups**						
Vegetables (g/d)	179.6 (99.55)	284.3 (129.4)*	175.4 (101.4)	293.4 (129.8)*	265.01 (114.1)	166.8 (109.1)*
Fruits (g/d)	260.5 (160.9)	458.8 (228.8)*	254.5 (151.6)	476.6 (236.7)*	392.4 (211.6)	292.7 (216.4)*
Legumes (g/d)	26.3 (16.31)	46.33 (45.77)*	26.21 (16.55)	47.42 (48.69)*	41.08 (31.60)	30.37 (41.88)*
Cereal grains	76.63 (35.66)	112.6 (54.56)*	85.75 (41.99)	102.8 (57.0)*	81.54 (43.07)	100.5 (46.80)*
Wholegrains (g/d)	6.29 (12.94)	16.61 (22.64)*	3.81 (9.01)	20.65 (24.40)*	16.61 (20.21)	5.12 (14.88)*
Refined grains (g/d)	70.33 (34.44)	95.97 (53.22)*	81.94 (41.44)	82.18 (52.21)	64.93 (39.53)	95.34 (43.38)*
Nuts (g/d)	3.18 (4.60)	8.90 (19.51)*	2.73 (3.92)	9.86 (11.03)*	7.62 (8.45)	2.87 (6.28)*
Olive oil (g/d)	14.93 (10.96)	22.59 (14.15)*	12.76 (9.18)	24.81 (15.13)*	19.72 (12.35)	17.87 (13.68)
Potatoes (g/d)	22.25 (20.97)	38.92 (24.22)*	28.23 (20.83)	30.28 (23.79)	26.0 (23.6)	35.6 (23.5)*
Fruit juices (g/d)	63.58 (81.96)	52.37 (60.02)	72.21 (83.10)	49.80 (72.82)*	38.09 (46.91)	79.90 (87.32)*
Dairy products (g/d)	586.7 (245.8)	394.5 (214.0)*	516.5 (248.8)	439.3 (234.4)*	475.1 (221.1)	532.5 (264.0)*
Eggs (g/d)	20.32 (9.85)	19.76 (11.55)	19.98 (8.06)	19.76 (11.08)	22.34 (9.70)	17.19 (8.66)*
Fish and Seafood (g/d)	33.93 (16.08)	34.53 (16.87)	31.69 (16.03)	37.02 (17.74)*	41.14 (16.80)	26.13 (13.66)*
Meat (g/d)	148.1 (46.27)	117.8 (42.55)*	139.9 (43.11)	118.9 (46.36)*	131.3 (46.56)	134.9 (44.50)
Animal fat (g/d)	1.11 (2.15)	0.49 (1.43)*	1.01 (1.88)	0.70 (1.79)	1.08 (2.09)	0.54 (1.48)*
Sugar-sweetened beverages (g/d)	12.51 (24.14)	10.92 (19.43)	18.37 (29.49)	7.16 (17.26)*	5.89 (12.04)	20.23 (33.69)*
Pastries and sweets (g/d)	40.08 (26.48)	34.27 (18.64)*	42.79 (26.32)	32.96 (26.72)*	29.24 (16.37)	50.83 (33.67)*
Miscellaneous food^1^ (g/d)	49.68 (24.76)	45.33 (20.91)*	52.76 (23.64)	40.71 (21.30)*	42.95 (19.04)	51.46 (24.63)

*TEI* Total Energy Intake, *FP* food pattern, *g/d* grams/days, *T1* tertile 1, *T2* tertile 2, *T3* tertile 3, *IQR* Interquartile range, *SFA* saturated fatty acids, *MUFA* monounsaturated fatty acids, *PUFA* polyunsaturated fatty acids

^1^Miscellaneous food includes pizza, lasagna, instant soup, mayonnaise, croquettes, nuggets, snacks, popcorn

^*^p for trend < 0.05

We calculated the energy-adjusted mean intake for each micronutrient in each tertile of the three scores (Table 4). In the overall provegetarian FP, participants in T3 had higher intakes of vitamins A, C, E, B1, and B6, and folate, Fe, Mg, Cr, and K, compared to children in T1. Conversely, they had lower intakes of vitamins D, B2, and B12, and Ca, I, P, Zn and Na. Regarding the healthful provegetarian FP, participants in T3 had higher intakes of vitamins A, C, E, B1, B2, B3, B6, folate, Fe, P, Mg, Cr and K, compared to those in T1. On the contrary, they displayed lower intakes of vitamin B12, Ca, I, Se, Zn and Na. Despite these lower intakes, participants in the highest tertile of the healthful provegetarian FP score did not exhibit a higher prevalence of inadequacy of any micronutrient, compared to children in T1. Lastly, regarding the unhealthful provegetarian FP, participants in T3 had lower intakes of all micronutrients and higher prevalence of micronutrient inadequacy for vitamins A, C, and E, and folate, Ca, I, and K, compared to children in T1.

**Table 4:** Energy-adjusted intake of micronutrients by tertiles of overall, healthful, and unhealthful provegetarian FP scores. Mean (SD)

	Overall provegetarian FP		Healthful provegetarian FP		Unhealthful provegetarian FP	
	T1	T3	T1	T3	T1	T3
Score Range	19–34	39–52	31–48	55–71	33–48	55–70
*n*	354	282	323	247	303	250
Vitamin A (equiv Retinol) (µg/d)	988.5 (22.96)	1159 (25.77)*	959.7 (23.89)	1205 (27.29)*	1201 (24.23)	887.3 (26.77)*
Vitamin C (mg/d)	113.3 (2.97)	172.9 (3.34)*	114.9 (3.11)	178.9 (3.56)*	162.0 (3.32)	108.4 (3.67)*
Vitamin D (µg/d)	3.14 (0.09)	3.11 (0.10)	3.08 (0.10)	3.14 (0.11)	3.55 (0.10)	2.56 (0.11)*
Vitamin E (mg/d)	7.72 (0.14)	9.39 (0.15)*	7.69 (0.14)	9.61 (0.16)*	9.05 (0.15)	7.32 (0.17)*
Vitamin B1 (mg/d)	1.40 (0.01)	1.47 (0.01)*	1.40 (0.01)	1.47 (0.01)*	1.49 (0.01)	1.35 (0.01)
Vitamin B2 (mg/d)	2.16 (0.02)	1.88 (0.03)*	2.04 (0.03)	1.99 (0.03)*	2.16 (0.03)	1.90 (0.03)
Vitamina B3 (mg/d)	35.97 (0.40)	35.37 (0.45)	34.97 (0.42)	36.16 (0.48)*	38.89 (0.40)	31.37 (0.44)*
Vitamin B6 (mg/d)	2.20 (0.02)	2.43 (0.03)*	2.15 (0.02)	2.48 (0.03)*	2.52 (0.02)	2.03 (0.03)*
Folic Acid (µg/d)	272.0 (3.85)	337.1 (4.32)*	273.3 (4.06)	341.3 (4.63)*	327.1 (4.23)	263.5 (4.68)*
Vitamin B12 (µg/d)	5.03 (0.07)	4.13 (0.07)*	4.76 (0.07)	4.42 (0.08)	5.13 (0.07)	4.12 (0.08)*
Ca (mg/d)	1247 (12.35)	1076 (13.86)*	1188 (13.59)	1127 (15.52)	1208 (13.91)	1118 (15.37)
I (µg/d)	117.6 (1.10)	98.69 (1.24)*	110.8 (1.24)	105.0 (1.42)	115.7 (1.24)	100.7 (1.37)*
Fe (mg/d)	13.19 (0.11)	14.66 (0.12)*	13.25 (0.11)	14.74 (0.13)*	14.70 (0.12)	12.89 (0.13)*
P (mg/d)	1718 (33.90)	1699 (38.05)	1643 (35.55)	1784 (40.61)*	1819 (36.44)	1594 (40.26)
Mg (mg/d)	285.7 (2.29)	318.9 (2.57)*	279.1 (2.27)	329.5 (2.60)*	322.1 (2.34)	270.3 (2.59)
Se (µg/d)	71.90 (0.70)	71.26 (0.78)	71.63 (0.73)	70.87 (0.84)*	72.76 (0.75)	69.72 (0.83)
Zn (mg/d)	9.87 (0.10)	9.29 (0.12)	9.41 (0.11)	9.60 (0.13)*	10.31 (0.11)	8.79 (0.12)*
Cr (µg/d)	64.44 (1.06)	70.38 (1.19)*	62.99 (1.10)	72.40 (1.26)*	72.28 (1.12)	59.84 (1.24)*
K (mg/d)	3236 (31.32)	3628 (35.15)*	3173 (31.89)	3734 (36.43)*	3685 (32.21)	3043 (35.58)*
Na (mg/d)	2990 (44.71)	2816 (50.18)	2926 (46.83)	2769 (53.49)	2927 (48.62)	2850 (53.71)*

^*^p for trend < 0.05

**Fig. 1:**
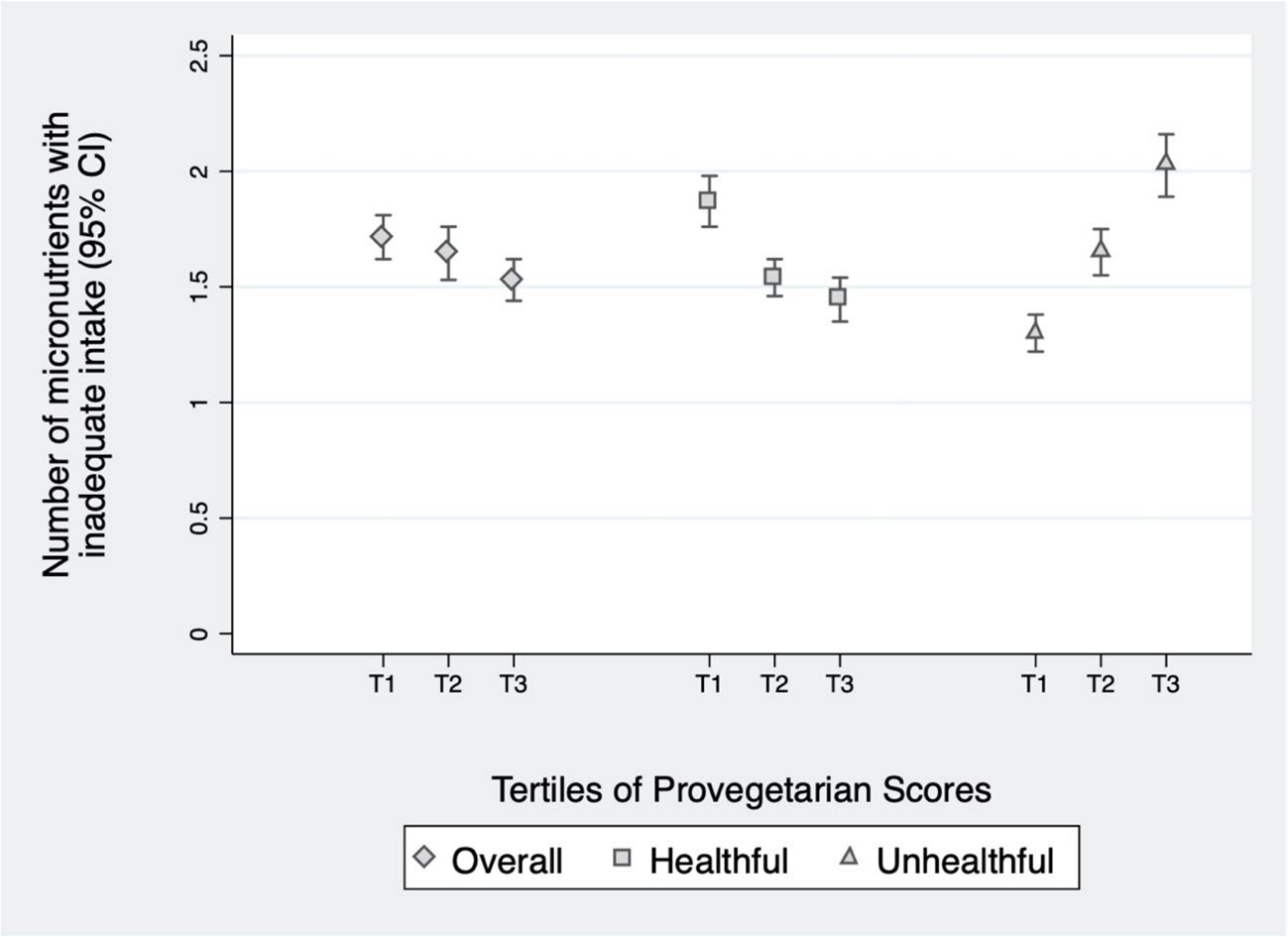
Number of micronutrients with inadequate intake (95% CI) in each tertiles of the overall, healthful, and unhealthful provegetarian FP scores. Adjusted for sex, age, energy intake, breastfeeding duration, number of siblings, parental knowledge about nutritional recommendations for children, parental attitudes towards child’s dietary habits, physical activity, and screen time. **Number of micronutrients with inadequate intake: Overall provegetarian FP** : T1: 1.71% (95% CI: 1.62%-1.81%), T2 : 1.65% (95% CI: 1.53%-1.76%), T3 : 1.53% (95% CI: 1.44%-1.62%). **Healthful provegetarian FP** : T1: 1.87% (95% CI: 1.76%-20.9%), T2 : 1.54% (95% CI: 1.46%-1.62%), T3 : 1.45% (95% CI: 1.35%-1.54%). **Unhealthful Provegetarian FP** : T1: 1.30% (95% CI: 1.22%-1.38%), T2: 1.65% (95% CI; 1.55%-1.75%); T3 : 2.03% (95% CI: 1.89%-2.16%)

After adjusting for potential confounders, we observed that the number of micronutrients with unmet EAR in each tertile decreased as the overall provegetarian FP score improved (p < 0.001) (Fig. 1). With the healthful provegetarian FP, the tendency was more pronounced. Conversely, the number of inadequate intakes of micronutrients rises as adherence to the unhealthful provegetarian FP increases.

Table 5 shows a significant linear trend in the odds of having ≥ 3 inadequate micronutrient intake across tertiles of the healthful provegetarian FP score. In the most adjusted model, participants in T3 had 0.47-fold lower odds (95%CI: 0.23–0.95) of having ≥ 3 inadequate intakes than those in T1 (p for trend = 0.015). An opposite trend could be observed for the unhealthful provegetarian FP score: participants in T3 had 20.06 higher odds (95%CI 9.2%-43.8%) of having ≥ 3 inadequate intakes of micronutrients (p for trend < 0.001) than their peers in T1.

**Table 5:** Odds Ratio and 95%CI for inadequate intake of ≥ 3 micronutrients associated with tertiles of overall, healthful, and unhealthful provegetarian FP scores

	OR (95% CI)			
**Overall provegetarian score**	**T1**	**T2**	**T3**	***p* for trend**
Score range	19–34	35–38	39–52	
% of participants with ≥ 3 inadequate intakes of micronutrients	18.36	14.03	10.63	
Crude	1.00 (ref)	0.72 (0.46–1.14)	0.52 (0.33–0.83)	0.006
Multivariate adjusted model 1	1.00 (ref)	0.71 (0.40–1.25)	0.79 (0.43–1.42)	0.356
Multivariate adjusted model 2	1.00 (ref)	0.69 (0.38–1.26)	0.84 (0.44–1.60)	0.495
Multivariate adjusted model 3	1.00 (ref)	0.70 (0.38–1.30)	0.90 (0.47–1.71)	0.623
**Healthful provegetarian score**	**T1**	**T2**	**T3**	***p* for trend**
Score range	31–48	49–54	55–71	
% of participants with ≥ 3 inadequate intakes of micronutrients	24.77	9.86	7.29	
Crude	1.00 (ref)	0.34 (0.22–0.53)	0.24 (0.13–0.54)	< 0.001
Multivariate adjusted model 1	1.00 (ref)	0.44 (0.25–0.79)	0.40 (0.21–0.77)	0.002
Multivariate adjusted model 2	1.00 (ref)	0.47 (0.26–0.86)	0.45 (0.22–0.90)	0.011
Multivariate adjusted model 3	1.00 (ref)	0.48 (0.27–0.87)	0.47 (0.23–0.95)	0.015
**Unhealthful provegetarian score**	**T1**	**T2**	**T3**	**p for trend**
Score range	33–48	49–54	55–70	
% of participants with ≥ 3 inadequate intakes of micronutrients	5.94	16.40	23.20	
Crude	1.00 (ref)	3.19 (1.82–5.59)	5.04 (2.88–8.83)	< 0.001
Multivariate adjusted model 1	1.00 (ref)	5.37 (2.71–10.63)	20.50 (9.85–42.68)	< 0.001
Multivariate adjusted model 2	1.00 (ref)	5.41 (2.63–11.11)	20.05 (9.17–43.82)	< 0.001
Multivariate adjusted model 3	1.00 (ref)	5.42 (2.63–11.16)	20.06 (9.19–43.79)	< 0.001

Model 1: adjusted for sex (male vs. female), age (continuous), and energy intake (continuous);

Model 2: additionally adjusted for number of children (1, 2, 3–4, 5 or more), breastfeeding duration (none, < 6 months, 6–12 months, and > 12 months), parental knowledge about nutritional recommendations (low, medium, and high score) for children, and parental attitudes towards child’s dietary habits (low, medium, and high score);

Model 3: additionally adjusted for moderate–vigorous physical activity (continuous) and screen time (continuous)

**Fig. 2:**
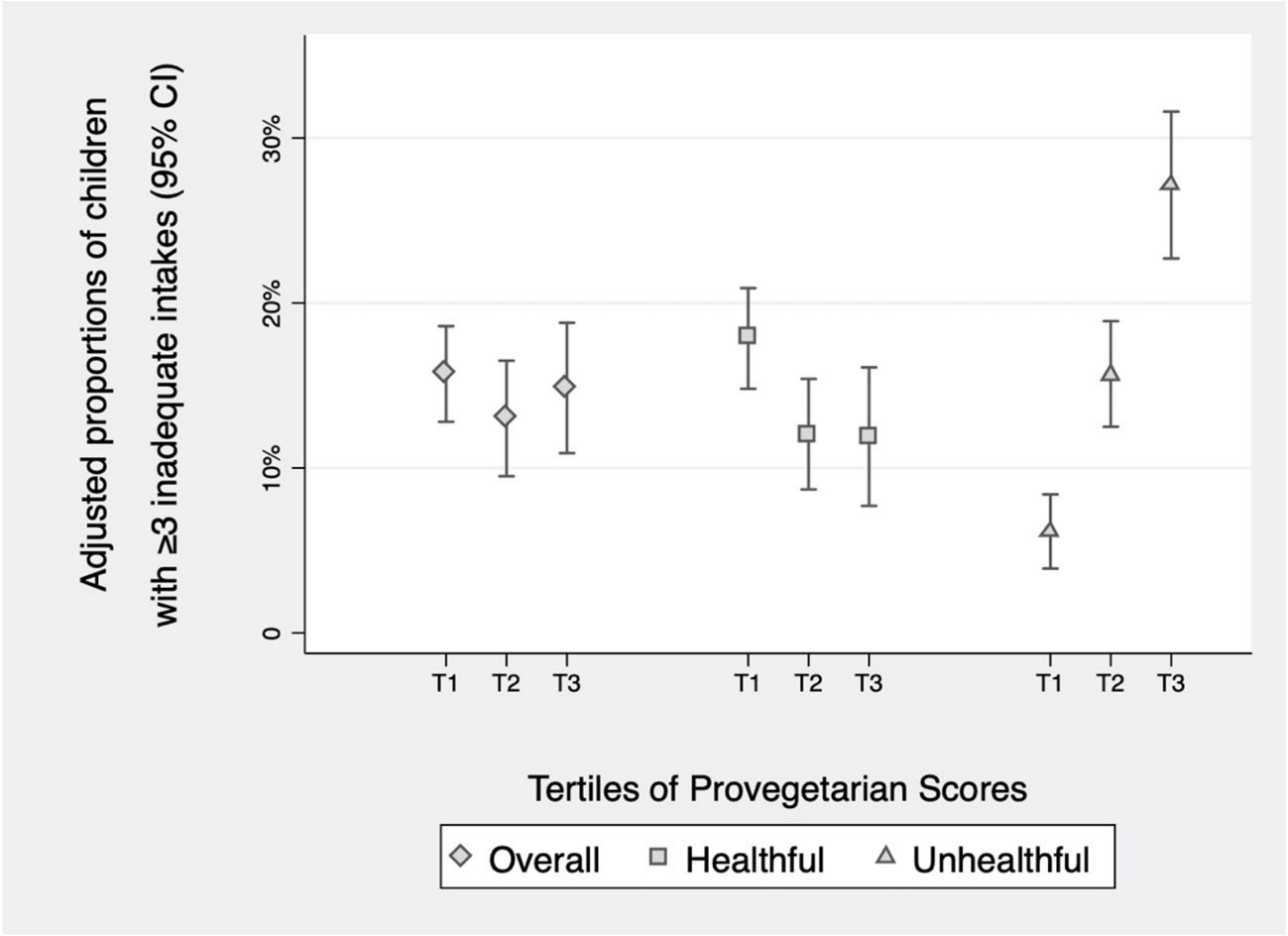
Adjusted proportions of children with ≥ 3 inadequate micronutrient intake (95% CI) in each tertile of the overall, healthful, and unhealthful provegetarian FP scores. Adjusted for sex, age, energy intake, breastfeeding duration, number of siblings, parental knowledge about nutritional recommendations for children, parental attitudes towards child’s dietary habits, physical activity and screen time. **Adjusted proportions of children with ≥ 3 inadequate intakes: Overall provegetarian FP** : T1: 15.7% (95% CI: 12.8%-18.6%), T2 : 13.0% (95% CI: 9.5%-16.5%), T3 : 14.8% (95% CI: 10.9%-18.8%). **Healthful provegetarian FP** : T1: 17.9% (95% CI: 14.8%-20.9%), T2 : 12.0% (95% CI: 8.7%-15.4%), T3 : 11.9% (95% CI: 7.7%-16.1%). **Unhealthful Provegetarian FP** : T1: 6.1% (95% CI: 3.9%-8.4%), T2: 15.6% (95% CI; 12.5%-18.9%); T3 : 27.1% (95% CI: 22.7%-31.6%)

The adjusted proportions of children with inadequate intake of ≥ 3 micronutrients in each tertile of the three indexes are presented in Fig. 2**.**

Finally, these results remained significant after performing two sensitivity analyses that accounted for supplement use (**Table S4**) and explored the association of provegetarian FP scores with the odds of not meeting the EAR for ≥ 4 micronutrients.

## Discussion

In this cross-sectional study of Spanish preschoolers we analyzed the association between provegetarian FPs and micronutrient adequacy by using an overall provegetarian FP score and two others to distinguish between healthful and unhealthful provegetarian FPs. We did not find a significant association between the overall provegetarian FP and micronutrient adequacy, though the point estimates suggested that a higher adherence to the overall provegetarian FP was associated with lower risk of inadequacy. However, we did observe an inverse association between the healthful provegetarian FP score and the number of micronutrients with unmet EAR. On the other hand, a harmful association was observed for the unhealthful provegetarian FP: higher adherence was associated with higher prevalence of micronutrient inadequacy.

Overall, our findings add evidence in favor of provegetarian FPs when they are based on healthy plant-based foods. On the contrary, the sole elimination of animal-derived foods without improving the quality of plant foods appears to be detrimental to micronutrient adequacy. In line with this, previous research has shown that low-quality diets, particularly those high in ultra processed foods, can compromise nutrient adequacy in children [26].

Notably, children with higher adherence to the healthful provegetarian FP displayed higher intakes of all micronutrients except for vitamin B12, Ca, I, Se, and Zn. It has been previously reported that consuming these micronutrients in sufficient amounts can prove to be challenging when decreasing the intake of animal foods [8, 9, 27, 28]. A recent study conducted in adults found that a greater adherence to a healthful provegetarian FP negatively affected the intakes of those same micronutrients [14]. In line with this, several authors have expressed concerns that the EAT-Lancet reference diet, a planetary flexitarian diet with low targets for animal product intake, may fall short in a similar range of micronutrients (vitamins B12 and D, Ca, I, Se, and Zn) both in adults [29, 30] and children [31]***.*** This is particularly relevant since these critical nutrients are consumed in insufficient amounts worldwide, both by adults and children [32–34]. In addition, experts have pointed out that the high content in phytate of plant-based diets could further affect their status by hindering their absorption [35].

In our study, despite lower intakes of vitamin B12, Ca, I, Se, and Zn in the group with highest adherence to the healthful provegetarian FP, this did not translate to a decrease in adequacy for any micronutrient (Table 4). Nonetheless, it is important to highlight that our participants, even in the highest tertiles of all three patterns, still consumed animal-derived products in amounts that significantly exceeded the dietary targets defined by the EAT-Lancet diet [1], even if their average intake was overall lower than those reported in population-based studies in Spain [36]. These findings indicate that there is room for reduction of animal food consumption without harming nutritional adequacy in our population of preschoolers, and presumably in other similar populations with equal or higher consumption of animal-derived foods. Future studies with a broader range of provegetarian FPs among participants might further elucidate the question and provide evidence to address these concerns in future dietary guidelines.

We acknowledge that our study has limitations. First, our study is based on self-reported information which could come with measurement errors. FFQ tend to overestimate food intake, and this extends to nutrient intake, which could have led to underestimate actual micronutrient inadequacies in our participants. However, the FFQ used in this study has been previously validated [20] and we have excluded participants with extreme energy and micronutrient intakes in our analyses. Second**,** the participants of the SENDO cohort tend to present a better diet quality than that of the average Spanish children [37], which can be explained by the fact that our participants have highly educated parents and that most participants enrolled in cohort studies focused on nutrition tend to be more health conscious in general [38]. This particularity of our sample could have resulted in a lower number of nutrient inadequacies than those that might have shown up in other population-based studies. However, this does not in itself invalidate our conclusions as they should be generalized based on subjacent mechanisms and not on the representativeness of our sample [39]. Third, we have assessed probability of micronutrient adequacy but not actual micronutrient deficiency, which can be better assessed through biomarkers. Finally, given that our study is observational, we cannot exclude the possibility of residual confounding by unknown factors.

On the other hand, our study displays several strengths: to the best of our knowledge, this is first study to analyze the relation between provegetarian FPs and micronutrient adequacy in children. Secondly, its extensive questionnaire enabled us to adjust the data for many potential confounders. Thirdly, the FFQ has been previously validated [20]. Finally, we used GEE models to take into account the potential correlation between siblings, which is a common limitation of studies in pediatric populations.

In conclusion, our results show that adherence to a healthful provegetarian FP is associated with improved nutritional adequacy in young children. In contrast, following an unhealthful provegetarian FP proved significantly detrimental to micronutrient adequacy. Our findings suggest that the reduction of animal food consumption in pediatric populations with moderate intakes of animal foods, in the context of a healthy diet, can probably be carried without compromising micronutrient adequacy. However, our reliance on self-reported dietary data, the specific characteristics of our sample, and the focus on micronutrient adequacy rather than actual deficiency limit the generalizability of our findings. More research is needed to better understand the relationship between provegetarian FPs and micronutrient adequacy in children, with an emphasis on using biomarkers for more accurate assessment.

## Supplementary Information

Below is the link to the electronic supplementary material.

Supplementary file1 (DOCX 224 KB)

## Data Availability

Data is available on request.

## Abbreviations

AI: Adequate Intake
BMI: Body Mass Index
CI: Confidence Interval
EAR: Estimated Average Requirement
FFQ: Food-Frequency Questionnaire
FP: Food pattern
OR: Odds Ratio
SD: Standard Deviation
SENDO: Seguimiento del Niño para un Desarrollo Óptimo
T1: Tertile 1
T2: Tertile 2
T3: Tertile 3
TEI: Total Energy Intake
Ca: Calcium
I: Iodine
Fe: Iron
P: Phosphorous
M: Magnesium
Se: Selenium
Zn: Zinc
Cr: Chromium
K: Potassium
Na: Sodium
