# Supplementary material for: Association between provegetarian food patterns and micronutrient adequacy in preschoolers: the SENDO project
Source: Eur J Pediatr. 2024 Oct 7;183(12):5267–78. doi: 10.1007/s00431-024-05808-9 (PMC11527897; doi:10.1007/s00431-024-05808-9)

**Association between Provegetarian Food Patterns and Micronutrient Adequacy in Preschoolers: the SENDO project**

**SUPPLEMENTARY MATERIAL**

**Table S1. Food Groups Components**

| **Component** | **Included foods** |
| --- | --- |
| **Plant food groups** |  |
| 1. Vegetables | Carrot, swiss chard, cabbage, cauliflower, lettuce, tomatoes, green beans, eggplant, peppers, asparagus, spinach, eggplants, zucchinis, cucumber, leek, pumpkin, peppers, asparagus, onion, other fresh vegetables |
| 2. Fruit | Citrus, banana, apple, pear, strawberry, peach, cherry, plum, fig, melon, watermelon, grapes, kiwi, canned fruit, pineapple, avocado, mango |
| 3. Legumes | Lentils, chickpeas, beans, peas |
| 4. Cereals | White bread, whole-grain bread, cold breakfast cereal, rice, pasta |
| 5. Potatoes | French fries, boiled potatoes |
| 6. Nuts | Almonds, peanuts, hazelnuts, pistachios, pine nuts, walnuts |
| 7. Olive oil | Common (refined) olive oil, virgen olive oil, extra-virgin olive oil |
| **Animal food groups** |  |
| 8. Meats/meat products | Beef, pork, lamb, rabbit, liver, chicken, turkey, cooked ham, Parma ham, mortadella, salami, paté, foie gras, spicy pork sausage, bacon, cured meats, hamburger, hot dog |
| 9. Animal fats for cooking or as a spread | Butter, lard |
| 10. Eggs | Eggs |
| 11. Fish and other seafood | White fish, dark-meat fish, salad or smoked fish, clams, mussels, shrimp, squid |
| 12. Dairy products | Whole milk, skim or low-fat milk, cream, milk shake, yogurt, custard, cheese |

**Table S2. Prevalence of inadequate micronutrient intake according to tertiles of of overall, healthful, and unhealthful provegetarian FP scores. N (percentages)**

|  | Overall provegetarian FP | |  | Healthful provegetarian FP | |  | Unhealthful provegetarian FP | |
| --- | --- | --- | --- | --- | --- | --- | --- | --- |
|  | T1 | T3 |  | T1 | T3 |  | T1 | T3 |
| Score Range | 19-34 | 39-52 |  | 31-48 | 55-71 |  | 33-48 | 55-70 |
| n | 354 | 282 |  | 323 | 247 |  | 303 | 250 |
| % Inadequate Intake |  |  |  |  |  |  |  |  |
| Vitamin A (equiv Retinol) (µg/d) | 6 (1.7) | 0 (0.0) |  | 6 (1.9) | 0 (0.0) |  | 1 (0.3) | 3 (1.2) |
| Vitamin C (mg/d) | 2 (0.6) | 0 (0.0) |  | 2 (0.6) | 0 (0.0) |  | 0 (0.0) | 3 (1.2) |
| Vitamin D (µg/d) | 354 (100.0) | 282 (100.0) |  | 323 (100.0) | 247 (100.0) |  | 303 (100.0) | 250 (100.0) |
| Vitamin E (mg/d) | 125 (35.3) | 30 (10.6) |  | 129 (39.9) | 19 (7.7) |  | 60 (19.8) | 84 (33.6) |
| Vitamin B1 (mg/d) | 0 (0.0) | 0 (0.0) |  | 0 (0.0) | 0 (0.0) |  | 0 (0.0) | 0 (0.0) |
| Vitamin B2 (mg/d) | 0 (0.0) | 0 (0.0) |  | 0 (0.0) | 0 (0.0) |  | 0 (0.0) | 0 (0.0) |
| Vitamina B3 (mg/d) | 0 (0.0) | 0 (0.0) |  | 0 (0.0) | 0 (0.0) |  | 0 (0.0) | 0 (0.0) |
| Vitamin B6 (mg/d) | 0 (0.0) | 0 (0.0) |  | 0 (0.0) | 0 (0.0) |  | 0 (0.0) | 0 (0.0) |
| Folic Acid (µg/d) | 29 (8.2) | 2 (0.7) |  | 32 (9.9) | 2 (0.8) |  | 4 (1.3) | 19 (7.6) |
| Vitamin B12 (µg/d) | 0 (0.0) | 1 (0.4) |  | 0 (0.0) | 1 (0.4) |  | 0 (0.0) | 0 (0.0) |
| Ca (mg/d) | 46 (13.0) | 38 (13.5) |  | 65 (20.1) | 27 (10.9) |  | 29 (9.6) | 42 (16.8) |
| I (µg/d) | 12 (3.4) | 22 (7.8) |  | 23 (7.1) | 11 (4.5) |  | 7 (2.3) | 25 (10.0) |
| Fe (mg/d) | 0 (0.0) | 0 (0.0) |  | 0 (0.0) | 0 (0.0) |  | 0 (0.0) | 0 (0.0) |
| P (mg/d) | 0 (0.0) | 0 (0.0) |  | 0 (0.0) | 0 (0.0) |  | 0 (0.0) | 0 (0.0) |
| Mg (mg/d) | 0 (0.0) | 0 (0.0) |  | 0 (0.0) | 0 (0.0) |  | 0 (0.0) | 0 (0.0) |
| Se (µg/d) | 0 (0.0) | 0 (0.0) |  | 0 (0.0) | 0 (0.0) |  | 0 (0.0) | 0 (0.0) |
| Zn (mg/d) | 0 (0.0) | 0 (0.0) |  | 0 (0.0) | 0 (0.0) |  | 0 (0.0) | 0 (0.0) |
| Cr (µg/d) | 1 (0.3) | 0 (0.0) |  | 1 (0.3) | 0 (0.0) |  | 0 (0.0) | 1 (0.4) |
| K (mg/d) | 58 (16.4) | 15 (5.3) |  | 71 (22.0) | 8 (3.2) |  | 15 (5.0) | 47 (18.8) |
| Na (mg/d) | 2 (0.6) | 3 (1.1) |  | 2 (0.6) | 1 (0.4) |  | 0 (0.0) | 1 (0.4) |

*p for trend < 0.05

| **Tabla S3. Odds Ratio and 95%CI for inadequate intake of** ≥ **3 micronutrients associated with tertiles of overall, healthful, and unhealthful provegetarian scores after sensitivity analysis with supplements** | | | | | | | |  |  |  |  |  |  |  |
| --- | --- | --- | --- | --- | --- | --- | --- | --- | --- | --- | --- | --- | --- | --- |
|  |  | |  | |  | |  | |  | |  | | |  |
|  |  | |  | |  | |  | |  | |  | | |  |
|  | **OR (95% CI)** | | | | |  | |  | | | | |  | |
| **Overall Provegetarian Score** | **T1** | **T2** | | **T3** | | **p for trend** | |  | |  | |  |  |  |
| % of participants with ≥ 3 inadequate intakes of micronutrients | 17.75 | 15.07 | | 10.96 | |  | |  | |  | |  |  |  |
| Crude | 1.00 (ref) | 0.82 (0.53-1.26) | | 0.57 (0.36-0.91) | | 0.017 | |  | |  | |  |  |  |
| Multivariate adjusted model 1 | 1.00 (ref) | 0.73 (0.42-1.26) | | 0.75 (0.41-1.36) | | 0.320 | |  | |  | |  |  |  |
| Multivariate adjusted model 2 | 1.00 (ref) | 0.70 (0.39-1.25) | | 0.81 (0.42-1.59) | | 0.490 | |  | |  | |  |  |  |
| Multivariate adjusted model 3 | 1.00 (ref) | 0.72 (0.40-1.29) | | 0.87 (0.44-1.70) | | 0.603 | |  | |  | |  |  |  |
| **Healthful Provegetarian score** | **T1** | **T2** | | **T3** | | **p for trend** | |  | |  | |  |  |  |
| % of participants with ≥ 3 inadequate intakes of micronutrients | 24.02 | 10.24 | | 7.17 | |  | |  | |  | |  |  |  |
| Crude | 1.00 (ref) | 0.37 (0.24-0.58) | | 0.24 (0.14-0.41) | | <0.001 | |  | |  | |  |  |  |
| Multivariate adjusted model 1 | 1.00 (ref) | 0.44 (0.25-0.77) | | 0.36 (0.19-0.69) | | 0.001 | |  | |  | |  |  |  |
| Multivariate adjusted model 2 | 1.00 (ref) | 0.45 (0.25-0.83) | | 0.40 (0.20-0.79) | | 0.003 | |  | |  | |  |  |  |
| Multivariate adjusted model 3 | 1.00 (ref) | 0.46 (0.25-0.83) | | 0.41 (0.21-0.83) | | 0.004 | |  | |  | |  |  |  |
| **Unhealthful Provegetarian score** | **T1** | **T2** | | **T3** | | **p for trend** | |  | |  | |  |  |  |
| % of participants with ≥ 3 inadequate intakes of micronutrients | 6.17 | 16.20 | | 22.98 | |  | |  | |  | |  |  |  |
| Crude | 1.00 (ref) | 3.01 (1.75-5.15) | | 4.87 (2.82-8.40) | | <0.001 | |  | |  | |  |  |  |
| Multivariate adjusted model 1 | 1.00 (ref) | 5.35 (2.74-10.45) | | 21.15 (10.05-44.53) | | <0.001 | |  | |  | |  |  |  |
| Multivariate adjusted model 2 | 1.00 (ref) | 5.18 (2.56-10.46) | | 19.60 (8.98-42.75) | | <0.001 | |  | |  | |  |  |  |
| Multivariate adjusted model 3 | 1.00 (ref) | 5.22 (2.57-10.57) | | 19.71 (8.99-43.17) | | <0.001 | |  | |  | |  |  |  |

Model 1: adjusted for sex (male vs. female), age (continuous), and energy intake (continuous);

Model 2: additionally adjusted for number of children (1, 2, 3–4, 5 or more), breastfeeding duration (none, <6 months, 6–12 months, and >12 months), parental knowledge about nutritional recommendations (low, medium, and high score) for children, and parental attitudes towards child’s dietary habits (low, medium, and high score);

Model 3: additionally adjusted for moderate–vigorous physical activity (continuous) and screen time (continuous).

| **Tabla S4. Odds Ratio and 95%CI for inadequate intake of** ≥ **4 micronutrients associated with tertiles of overall, healthful, and unhealthful provegetarian scores (sensitivity analysis)** | | | | | | | |  |  |  |  |  |  |  |
| --- | --- | --- | --- | --- | --- | --- | --- | --- | --- | --- | --- | --- | --- | --- |
|  |  | |  | |  | |  | |  | |  | | |  |
|  |  | |  | |  | |  | |  | |  | | |  |
|  | **OR (95% CI)** | | | | |  | |  | | | | |  | |
| **Overall Provegetarian Score** | **T1** | **T2** | | **T3** | | **p for trend** | |  | |  | |  |  |  |
| % of participants with ≥ 4 inadequate intakes of micronutrients | 10.45 | 7.46 | | 3.55 | |  | |  | |  | |  |  |  |
| Crude | 1.00 (ref) | 0.68 (0.38-1.24) | | 0.33 (0.16-0.65) | | 0.001 | |  | |  | |  |  |  |
| Multivariate adjusted model 1 | 1.00 (ref) | 0.74 (0.35-1.54) | | 0.44 (0.18-1.08) | | 0.068 | |  | |  | |  |  |  |
| Multivariate adjusted model 2 | 1.00 (ref) | 0.73 (0.34-1.56) | | 0.47 (0.17-1.25) | | 0.119 | |  | |  | |  |  |  |
| Multivariate adjusted model 3 | 1.00 (ref) | 0.78 (0.36-1.69) | | 0.57 (0.21-1.56) | | 0.254 | |  | |  | |  |  |  |
| **Healthful Provegetarian score** | **T1** | **T2** | | **T3** | | **p for trend** | |  | |  | |  |  |  |
| % of participants with ≥ 4 inadequate intakes of micronutrients | 15.48 | 2.72 | | 2.43 | |  | |  | |  | |  |  |  |
| Crude | 1.00 (ref) | 0.16 (0.08-0.34) | | 0.14 (0.06-0.32) | | <0.001 | |  | |  | |  |  |  |
| Multivariate adjusted model 1 | 1.00 (ref) | 0.23 (0.10-0.54) | | 0.26 (0.09-0.76) | | 0.004 | |  | |  | |  |  |  |
| Multivariate adjusted model 2 | 1.00 (ref) | 0.26 (0.10-0.63) | | 0.29 (0.10-0.89) | | 0.007 | |  | |  | |  |  |  |
| Multivariate adjusted model 3 | 1.00 (ref) | 0.26 (0.11-0.65) | | 0.36 (0.12-1.10) | | 0.019 | |  | |  | |  |  |  |
| **Unhealthful Provegetarian score** | **T1** | **T2** | | **T3** | | **p for trend** | |  | |  | |  |  |  |
| % of participants with ≥ 4 inadequate intakes of micronutrients | 2.64 | 8.68 | | 11.60 | |  | |  | |  | |  |  |  |
| Crude | 1.00 (ref) | 3.35 (1.55-7.25) | | 4.85 (2.24-10.49) | | <0.001 | |  | |  | |  |  |  |
| Multivariate adjusted model 1 | 1.00 (ref) | 4.94 (1.92-12.71) | | 14.06 (5.04-39.22) | | <0.001 | |  | |  | |  |  |  |
| Multivariate adjusted model 2 | 1.00 (ref) | 5.18 (1.86-14.42) | | 14.23 (4.51-44.88) | | <0.001 | |  | |  | |  |  |  |
| Multivariate adjusted model 3 | 1.00 (ref) | 4.97 (1.80-13.68) | | 13.72 (4.43-42.48) | | <0.001 | |  | |  | |  |  |  |

Model 1: adjusted for sex (male vs. female), age (continuous), and energy intake (continuous);

Model 2: additionally adjusted for number of children (1, 2, 3–4, 5 or more), breastfeeding duration (none, <6 months, 6–12 months, and >12 months), parental knowledge about nutritional recommendations (low, medium, and high score) for children, and parental attitudes towards child’s dietary habits (low, medium, and high score);

Model 3: additionally adjusted for moderate–vigorous physical activity (continuous) and screen time (continuous).

**Fig.S1. Flow-chart of participants recruited in the SENDO project, 2014–2023.**


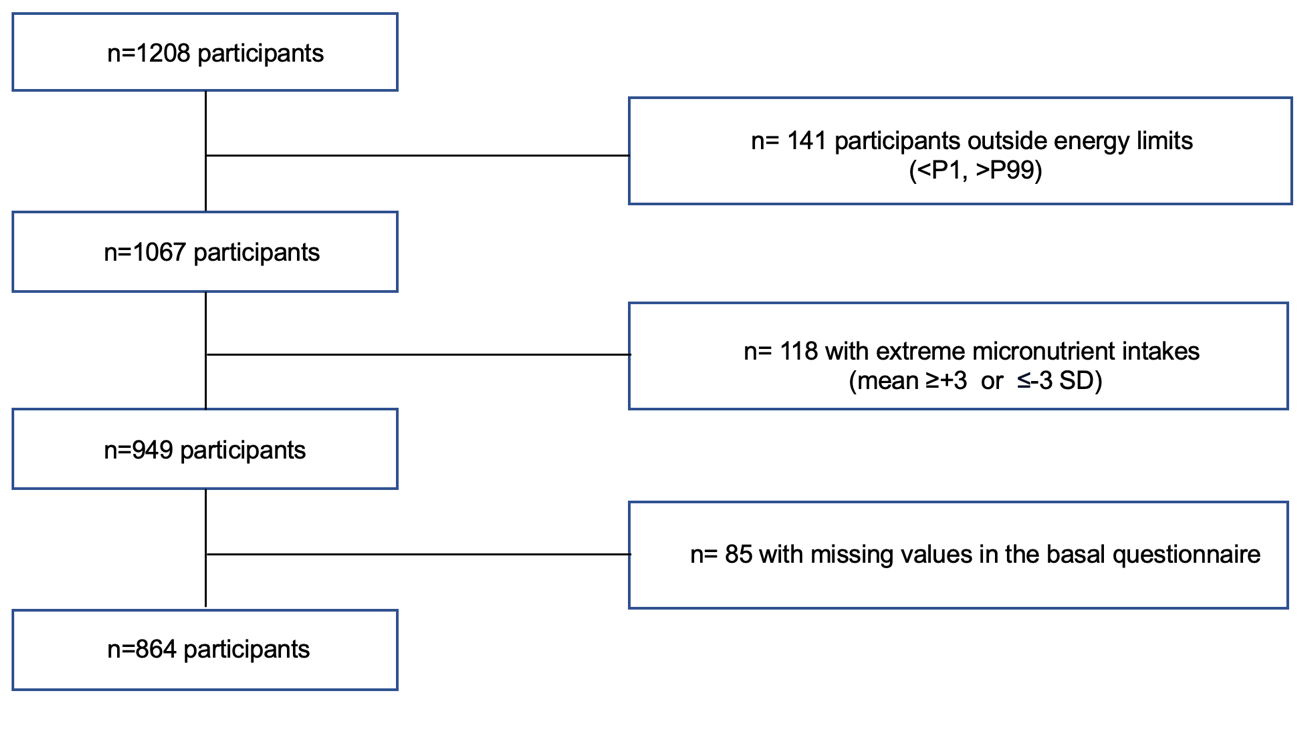

Supplement: Supplementary file 1 — Supplementary file1 (DOCX 224 KB) [file 431_2024_5808_MOESM1_ESM.docx]
